# Development of a secondary school-based digital behaviour change intervention to improve tooth brushing

**DOI:** 10.1186/s12903-021-01907-3

**Published:** 2021-10-22

**Authors:** Zoe Marshman, Sarab El-Yousfi, Ian Kellar, Donna Dey, Mark Robertson, Peter Day, Ivor Chestnutt, Sue Pavitt, Mariana de Araujo, Nicola Innes

**Affiliations:** 1 School of Clinical Dentistry, University of Sheffield, Claremont Crescent, Sheffield, S10 2TA UK; 2 School of Psychology, University of Leeds, Lifton Place, Leeds, LS2 9JT UK; 3 School of Education and Social Work, University of Dundee, Nethergate, Dundee, DD1 4HN UK; 4 School of Dentistry, University of Dundee, Park Place, Dundee, DD6 8EF UK; 5 School of Dentistry, University of Leeds, Leeds, LS2 9LU UK; 6 School of Dentistry, Cardiff University, Heath Park, Cardiff, CF14 4XY UK

**Keywords:** Oral health, Behaviour change, Text messages, Young people

## Abstract

**Background:**

Dental caries in adolescents remains a significant public health problem with few oral health promotion interventions aimed at reducing dental caries in secondary school-aged students. Previous oral health and mobile health (mHealth) research has suggested the need for the development of a school-based behaviour change intervention incorporating a digital component. This study aimed to describe the development process of a behaviour change intervention to improve the oral health of students aged 11–16 years attending secondary schools in the UK.

**Methods:**

A six-step process was used to develop the complex intervention informed by behaviour change theory and involving students, young people, parents and teachers in the process. The steps were: (1) identifying the target behaviours, namely tooth brushing with a fluoride toothpaste (2) identifying the theoretical basis and developing the causal model (3) reviewing the relevant literature and developing the logic model (4) designing the intervention with young people, parents and school staff (5) specifying the intervention content and (6) translating this content into features of the intervention and piloting.

**Results:**

The resultant intervention included a quality-assured classroom-based session (CBS) (guided by a lesson plan and teaching resources), delivered by school teachers which was embedded within the school curriculum. This CBS was followed by a series of (Short Message Service) SMS texts delivered twice daily to student’s mobile telephones with the content, duration and timing of the messages informed by involvement of students and young people.

**Conclusions:**

An intervention to improve the oral health of secondary school students through improved tooth brushing was rigorously developed based on behaviour change theory and work with young people, parents and school staff. Further research is needed to evaluate the outcomes and processes involved following the delivery of this intervention.

*BRIGHT Trial Trial Registration* ISRCTN12139369.

## Background

Dental caries in adolescents remains a significant public health problem, particularly in social and economically deprived areas [1]. The current focus of community oral health promotion interventions in the UK is to reduce dental caries mainly with children under 11 years of age [2]. Few interventions are aimed at reducing dental caries in adolescents despite this being a critical stage where health practices are developed [3]. Examples of current oral health promotion interventions to improve the oral health of adolescents have been categorised into oral health education interventions and more complex interventions involving additional activities such as clinical prevention measures alongside the education component [4]. However, limitations of existing research include a lack of understanding of factors influencing the oral health behaviours of adolescents has been and little is known about adolescent’s receptiveness to interventions that seek to change these behaviours [5]. Previous research has looked at short-term changes in behaviour only [6] with further research recommended to develop interventions based on behaviour change theory including long term evaluation [4].

Mobile health (mHealth) interventions are increasingly being used to bring about health behaviour change. mHealth describes multimedia technologies that interface with health care delivery, most commonly through mobile phones. Short message service (SMS) interventions are the most widely studied mHealth intervention [7]. A recent systematic review of SMS found a small but statistically significant weighted mean effect size for the impact of SMS on preventive health behaviour change (d = 0.24) with positive effect of SMS interventions in 11 of the 35 included studies, with a further 13 studies having mixed effects [8]. Few mHealth interventions have been developed to improve the oral health of young patients or people.

A study of unemployed young people in New Zealand, called ‘Keep on Brushing (KOB)’ investigated a weekly SMS and free toothbrushes and toothpaste programme [9]. The intervention was underpinned by the Health Belief Model and aimed to improve tooth brushing frequency among those aged 18–24 years. The study found self-reported tooth brushing of twice or more per day increased from 51% at baseline to 70% at week three, 74% at week six, and 73% at week nine. No important differences were noted between age, gender, or ethnic groups, although attrition was relatively high with only 26% participating by week nine. The authors concluded that motivational text messaging improved the self-reported oral health of this hard-to-reach group and suggested a randomised control trial was needed including a longer intervention co-produced with the target group.

The BRIGHT Trial: *B*rushing *R*em*I*nder 4 *G*ood oral *H*eal*T*h is a multi-centre, school-based, assessor-blinded, two-arm cluster-randomised control trial based on KOB [10]. BRIGHT aims to investigate the clinical and cost-effectiveness of a behaviour change intervention to improve the oral health of secondary school students (11–16 years) living in deprived areas of the UK. The BRIGHT trial also includes a process evaluation. The intervention is a multi-component, complex intervention with two parts; (1) a classroom-based session (CBS) embedded in the school curriculum and (2) a series of follow-up SMS text messages to student’s mobile telephones. BRIGHT includes around 40 schools with above the national average percentage of students eligible for free school meals. From these schools the trial has recruited over 4000 students aged 11–13 years. The primary outcome of the BRIGHT trial is the prevalence of obvious dental caries experience at 30 months.

Understanding the components of any complex intervention, their provenance and how they were derived has been recognised as being important in placing context around study findings and insight into their generalisabilty to other settings. It also allows potential users of the research to decide whether adaptations should be made for different circumstances. Yet reporting how complex interventions are developed and the theory underpinning them is often poor or even neglected [11, 12]. The aim of this paper is to describe the development process of a behaviour change intervention that is being used in the BRIGHT trial and the theory underlying its various components.

## Method

### Background to the intervention

The development of the intervention was informed by the guidance on the development of complex interventions [13] and behaviour change interventions [14] and designed *with* young people and other stakeholders. It is reported according to the GUIDED guideline for reporting of intervention development studies [12]. The intervention development framework employed was a combination approach, utilising both a theory and evidence-based approach, and a partnership approach [15].

### Setting for the intervention

Secondary schools were chosen as an appropriate setting for the intervention as they provide an opportunity to reach large numbers of young people at low cost [16]. The modes of delivery, CBS and SMS were chosen to be practicable, acceptable, safe, affordable, sustainable and equitable as recommended for behaviour change interventions [17].

### Intervention components

#### Classroom-based session (CBS)

The KOB study did not include any classroom activities so a bespoke lesson plan and teaching resources for the CBS were designed specifically for the BRIGHT trial. The CBS was developed to be delivered by teachers as part of the Personal Health and Social Education curriculum (England and Wales) and Health and Wellbeing (Scotland).

#### Short messaging service (SMS)

Key features of SMS health interventions include their duration and timing, tailoring them to the audience and linking SMS use with other activities, which in this case is the CBS [18]. However, limitations of using SMS include the restriction in the number of characters available, the need for basic literacy and limited access to mobile phones for some young people [19].

The results section will describe the six steps in the BRIGHT intervention development process:

- *Step 1* Identify target behaviours.
- *Step 2* Identify the theoretical base.
- *Step 3* Review relevant literature.
- *Step 4* Design the intervention with students, parents and school staff.
- *Step 5* Specify the intervention content.
- *Step 6* Translation of the intervention content into interventions features and piloting.

## Results

The intervention was developed using the following six steps:

*Step 1* Identify target behaviours.

The first step was to identify the target behaviour to reduce the prevalence of caries in permanent teeth. One of the most effective ways of reducing the prevalence of caries is twice-daily tooth brushing with fluoride toothpaste [20, 21]. Observational studies have shown the efficacy, frequency and duration of tooth brushing to be inadequate [22, 23] increasing the risk of caries [24, 25]. Behaviour change approaches are recommended to improve tooth brushing as a health behaviour [26]. The target behaviour was therefore improving the efficacy and frequency of tooth brushing with a fluoride toothpaste. Other behaviours to reduce dental caries related to diet were not targeted.

*Step 2* Identify the theoretical base.

**Fig. 1:**
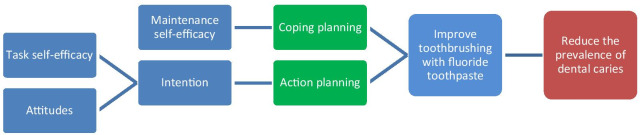
Causal model for the intervention

The intervention development drew on the Health Action Process Approach (HAPA) [27] as the causal model (Fig. 1) and was informed by the Behaviour Change Wheel [17].The main principles of the HAPA are that the health behaviour change process includes first a motivation phase (indicated in blue in Fig. 1) in which people develop their intentions, followed by a volitional phase. In the volitional phase action planning and coping planning (indicated in green) are needed to plan when, where, and how a behaviour will be conducted and for the anticipation of barriers to the behaviour.

*Step 3* Review relevant literature.

In addition to the theoretical base, the literature from the fields of oral self-care in adolescents, SMS and teaching young people about health was reviewed.

Systematic literature searches were conducted via electronic databases MEDLINE via Ovid, PsycINFO, Scopus and Google Scholar. Multiple combinations of search terms included *oral health, dental health, oral hygiene, oral self-care, tooth brushing, adolescents, children, behaviour change, intervention*, and *psychological.* Additional articles were identified through hand searching of reference lists of relevant articles. The searches focused on children aged 11–16 years. An existing relevant systematic review was identified of psychosocial factors considered important for oral hygiene behaviour in young people aged 9–19 years [28], the following factors were found to be influential:

Motivational factors

- Both maintenance- and task-*self-efficacy* refer to an individual’s belief in his or her ability to accomplish a task [29], such as effective tooth brushing.
- *Attitude *refers to the extent to which an individual perceives a particular (oral health) behaviour as favourable or unfavourable [30] based on the outcome expectancy and risk perceptions.
- *Intention* summarises an individual’s motivation to act [31].

Volitional factors

- *Action planning* involves moving beyond behavioural intentions because it includes the parameters of ‘when’, ‘where’ and ‘how’ [27] to practice oral hygiene behaviours. Explicitly specifying these parameters increases the likelihood that the behaviour will become habitual or automatic [32].
- *Coping planning* is a self-regulatory strategy where individuals anticipate possible barriers to a behaviour (such as tooth brushing) and devise coping strategies to overcome them [27].

These were consistent with the HAPA model [27]. Scheerman and colleagues also acknowledged that there may be other influences on tooth brushing behaviour in adolescents that have not previously been researched, including ‘self-determination’, ‘anticipated regret’, ‘action control’ and ‘self-identity’ [28]. Further investigation of these additional influences suggested there was enough evidence for ‘self-determination’ to be included as a general approach incorporated into the intervention. Self-determination is the degree to which an individual's behaviour is self-motivated. For behaviours such as tooth brushing which may be an activity that young people are uninterested in, with intangible benefits, an intervention needs to encourage them to value the outcomes of tooth brushing, to take responsibility for it and be competent to facilitate self-motivation [33, 34]. These factors were built into the intervention development, particularly for the CBS lesson plan and teaching resources.

To create the lesson plan and teaching resources for the CBS, first the curricula for England, Scotland and Wales were analysed including: science key stage 3 (a) and 4 (b) [35, 36]; Personal, Social, Health and Economic study key stage 3 (PSHE 2014), the Scottish Curriculum for excellence experiences and outcomes for both science and health and wellbeing [37, 38]; and the Welsh Personal and Social Education framework [39]. The literature suggested the CBS must be based on dialogue, perceived to be relevant by students, be understandable, trustworthy and positive. Furthermore, adolescents want active teaching with opportunities to discuss questions about lifestyle with others [40].

For the SMS, a search for systematic reviews was conducted to establish the optimal duration, frequency and content. A recent systematic review of SMS found the length of interventions typically ranged from 1 to 66 weeks with a median duration of 12 weeks. There was some suggestion that interventions lasting 6–12 months were associated with greater effects than shorter interventions. The frequency of messages varied from five times per day to once a month depending on the expected frequency of the targeted behaviour [18]. Most studies used tailored messages based on participant characteristics including age, gender or location and some personalised the messages. However, SMS design needs to take into account the possibility of annoyance, boredom, content blindness and potentially purposeful avoidance and mitigate for these [41]. It has been recommended that future studies ensure the intervention, including the SMS, was developed rigorously, that the SMS messages were suitable for the target population, tailored to individuals’ key characteristics such as their age and used the participant’s name. A systematic review of SMS interventions in adolescents highlighted the importance for young people of personal choice and that SMS should be positive, relevant, short and use informal language [42]. These considerations were factored into subsequent steps.

**Fig. 2:**
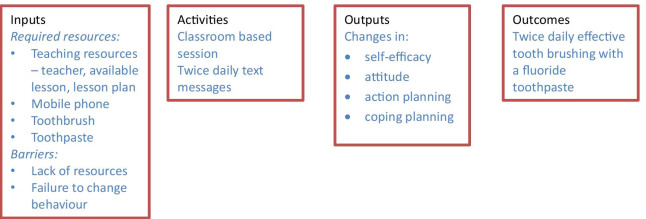
Intervention logic model

At the end of step 3 a logic model was developed (Fig. 2).

*Step 4* Design the intervention with students, parents and school staff.

To build on step 3 and partner with school staff, students and parents to co-produce the intervention, workshops were held in schools in England, Wales and Scotland. Forty-five students attended the workshop and discussions were held with a further fifteen young people in the BRIGHT trial youth forum run by the youth empowerment charity Chilypep. A workshop was also held with 14 parents and discussions were held with three school teachers and a lecturer in secondary education.

Next, the results of the workshops with students and parents were used to decide on the best approach to engage students of this age. The outcome of these workshops was the decision that the lesson should contain a series of learning tasks and a case study for students to consider barriers to tooth brushing and help them identify solutions to coping with such challenges.

Students described the aspects of tooth brushing of interest to them namely wanting clean teeth and fresh breath. They described that showing the ‘disgusting’ or ‘scary’ consequences of poor oral health was likely to motivate them to brush their teeth more often. Students had excellent knowledge of the need to brush teeth twice daily but wanted to know more facts about what causes dental caries, the benefits of tooth brushing and the consequences of not brushing. The literature review suggested that while children are very aware that sugar causes dental caries they do not appreciate the role brushing with a fluoride toothpaste plays in the prevention of dental caries. The first section of the lesson plan therefore included a teacher-led description of:

1. The reasons tooth brushing with fluoride toothpaste is important.
2. The consequences of not tooth brushing.

The students were then asked to identify for themselves the benefits of tooth brushing and the consequences of not brushing their teeth. The students in the workshops were able to identify the barriers that stopped them brushing, particularly in the evening. The principle barrier for students without parental control at bedtime was being ‘too busy’ to brush their teeth, particularly playing games or chatting to friends online. Other barriers at bed time included being too tired (and falling asleep or forgetting to brush) and being on a sleepover. In the morning, the main barrier was not wanting to get out of bed to brush their teeth.

Feedback from school stakeholders suggested the CBS should be developed so it could be delivered by teachers with no training required and all necessary resources, including a lesson plan, provided.

The outputs of the workshop were also used as the basis of the SMS. Students expressed a preference for simple messages, which did not come across as ‘nagging’, did not attempt at humour, were personalised and that were written in full text rather than ‘textspeak’. They requested some choice over the timing of the messages that would arrive in the morning and at night, including later choice of timing at weekends plus the ability to stop the messages if they became annoying.

Parents were positive about the intervention in terms of the potential for reducing dental caries and improving tooth brushing. Parents felt a CBS may be more effective at improving tooth brushing than their efforts at home. Parents suggested the wording of the SMS would be important for their effectiveness and wanted assurance of security of the telephone numbers.

*Step 5* Specify the intervention content.

Based on steps 2, 3 and 4 the components of the intervention were brought together. The behaviour change technique taxonomy (with associated codes provided in brackets) [43] was applied to select behaviour change technqiues based on the psychological determinants of tooth brushing behaviour (Table 1) and related to the causal model. This work predated the Theory and Technique Tool [44], but the behaviour change techniques selected are broadly supported by the links with mechanisms of action [45] constructs that relate to the determinants found in the causal model for this intervention (Table 1).

**Table 1:** Behaviour change techniques and description

Technique	Description
Information about health consequences (5.1)	The CBS explained the effectiveness of fluoride toothpaste at improving oral health based on concerns of students i.e. appearance, social reasons, health reasons including reducing dental caries This information was reinforced with the SMS
Goal setting (1.1)	The CBS encouraged the students to decide to improve their brushing (intention development) through a personalised brushing plan
Problem solving (1.2, 1.4)	The CBS helped students identify barriers and facilitators to tooth brushing and to develop personalised brushing plans (action and coping planning)
Instruction on how to perform a behaviour (4.1)	The CBS included a video clip and factsheet to show students how to brush effectively.This is re-enforced through the SMS to develop self-efficacy
Action planning (1.4)	The CBS involved detailed planning of what the student will do, including a definition of the behaviour, specifying twice daily tooth brushing for 2 min in terms of where, when and how
Prompts/cues (7.1)	The CBS taught students to identify cues (associated with times of day and transitionary spaces) that can be used to remind them to brush their teeth

The behaviour change technique codes are provided in brackets

*Step 6* Translation of the intervention content into interventions features and piloting.

The final step involved integrating the content through discussions between members of the research team, external experts and further refinement work with the BRIGHT youth forum, parents, school nurses and teachers.

Development of the CBS

This first section of the CBS aimed to develop student’s motivation to brush their teeth and stimulate sufficient reason for them to want to change their behaviour [46]. This content of the CBS was consistent with the motivational factors of the HAPA, which were further developed in the second section of the CBS, which focused on self-efficacy.

The second section focused on self-efficacy in terms of how to brush teeth well. The workshop stimulated several key questions students wanted to know including how to physically brush teeth well and a video clip was incorporated into the lesson plan to address this. Several questions arose from the workshops that students were interested in having answers to, these were incorporated into a Frequently Asked Questions (FAQ) fact sheet. The FAQ fact sheet was designed and produced by the BRIGHT youth forum.

The third section of the CBS focused on action planning, coping planning and peer support. Students in the workshops were able to identify the barriers that stopped them from tooth brushing but they found it more difficult to derive their own practical solutions that would be appropriate for the BRIGHT intervention. This finding led to the inclusion of a case study approach involving a character who students could relate to; ‘Charlie’ was faced with the kinds of barriers identified in the workshops. This persona work allowed the teacher to provide examples of the solutions that might work for Charlie. Following this group activity individual students were encouraged to identify their own barriers and solutions.

The lesson plan and resources were then quality assured by two teacher educators. Using this feedback, minor amendments were made. The lesson was then delivered as a pilot to a class of students aged 12–13 years of age. This opportunity allowed refinement of the resources and lesson plan. The teachers delivering the lesson confirmed all necessary materials were available for it to be delivered without the need for specific training.

Development of the content of SMS

The previous steps allowed a list of 50 candidate SMS messages to be drafted. These messages were reviewed by fifteen young people with each message rated on a 3-point scale (bad, OK, good). Only the messages that were considered OK or good by the majority of the young people were selected for piloting by the BRIGHT youth forum who received twice daily messages for two weeks and they were asked to identify which messages they preferred. Additionally, the youth forum suggested which times should be offered for morning text and evening texts during the weekday and weekends. A final schedule of 28 messages (2 per day over 14 days) was developed to be repeated until the student requested these to stop.

## Discussion

This paper has described the process through which a behaviour change intervention was developed using a theory and evidence-based approach, and a partnership approach [15] with the aim of improving the oral health of students aged 11–16 years attending secondary schools in the UK. The resultant intervention includes a CBS, delivered by school teachers which is re-enforced by a series of SMS messages delivered twice daily to student’s mobile telephones. The intervention development has been reported according to relevant guidelines [12].

The intervention was required to address the paucity of oral health promotion interventions for secondary school aged students (Public Health England, 2014) and attempted to integrate a traditional classroom-based delivery method complemented by a more novel mHealth technological solution. The strength of the intervention was its rigorous development based on behaviour change theory and designed *with* young people, parents and school staff. The CBS was developed to be embedded in the school curriculum which helped schools see the relevance of the topic and will facilitate future implementation. The SMS component was developed based on recommendations from previous studies on the need for the SMS to be appropriate for the target population, including diversity of messages to avoid annoyance, boredom and potentially content blindness [41].

However, as an intervention specifically designed to reduce dental caries, it has some limitations. First, the intervention was developed solely to focus on tooth brushing with a fluoride toothpaste and did not attempt to change other behaviours to reduce dental caries, specifically reduction of sugar consumption. This approach was chosen as the determinants of tooth brushing behaviours in young people are different to those related to diet and so the behaviour change techniques required are different. Further work is needed to develop effective, theory-driven oral health promotion programmes aimed at adolescents more generally.

Second, as stated in the logic model, the intervention requires students to have a mobile phone, a toothbrush and fluoride toothpaste. Providing toothpaste or toothbrushes as part of the intervention may be possible in some areas but would significantly influence the cost, particularly as the students would need regular supplies. One partial solution was letting students know, as part of the FAQ fact sheet, that cheap supermarket own brand oral hygiene products were available and as effective as branded products. While some students have limited access to mobile phones [19], there is also some research that suggests children with low socio-economic status have better mobile phone access than their more affluent peers [47].

The intervention is currently being evaluated through the BRIGHT randomised control trial which includes evaluation of (self-report and clinical) oral health outcomes and a mixed-methods process evaluation. The process evaluation plays an essential part in this trial, ensuring implementation, mechanisms of impact and context are assessed (Moore et al. 2015). Implementation will be explored in terms of the process through which the intervention (CBS and SMS) is delivered, what is delivered in different schools, the fidelity (consistency of delivery), dose (quantity of intervention delivered), reach (extent to which participants come into contact with intervention) and adaptations (alterations made to intervention for better contextual fit). The mechanisms of impact will be examined for how the intervention activities and student’s interactions with them trigger change in tooth brushing behaviours, self-efficacy, social norms, action and coping planning, self-determination and any unintended effects. Finally, context will be explored through examining the broader school culture and how it may have influenced and interacted with the delivery and functioning of the intervention and its outcomes. This includes external factors such as school structure and any changes to the curriculum. As a result of the outcome and process evaluation, further refinement of the intervention may be required.

## Conclusion

In conclusion, this intervention to improve the oral health of secondary school students through tooth brushing, was rigorously developed based on behaviour change theory and work with young people, parents and school staff. Further research is needed to evaluate the outcomes and processes involved following the delivery of this intervention.

## Data Availability

The datasets generated and analyzed during the current study are not publicly available as permission was not sought but are available from the corresponding author on reasonable request. The intervention is currently undergoing evaluation and the resources may require further refinement before they are finalised. The current drafts are available from the corresponding author.

## Abbreviations

mHealth: Mobile health
CBS: Classroom-based session
SMS: Short message service
KOB: Keep on brushing
HAPA: Health action process approach
PSHE: Personal, social, health and economic
FAQ: Frequently asked questions
BRIGHT: Brushing reminder 4 good oral health
